# Clinical Outcomes of Femtosecond Laser-assisted Implantation of 325-Degree Versus 340-Degree Arc Length Intracorneal Ring Segments in Naive Keratoconic Eyes

**DOI:** 10.18502/jovr.v18i4.14545

**Published:** 2023-11-30

**Authors:** Amir Faramarzi, Kiana Hassanpour, Marjan Mazouchi, Bahram Einollahi, Sepehr Feizi, Hamed Esfandiari, Mohammad-Mehdi Sadoughi, Majid Moshirfar

**Affiliations:** ^1^Ophthalmic Research Center, Research Institute for Ophthalmology and Vision Science, Shahid Beheshti University of Medical Sciences, Tehran, Iran; ^2^Department of Ophthalmology, Labbafinejad Medical Center, Shahid Beheshti University of Medical Sciences, Tehran, Iran; ^3^Department of Ophthalmology, Northwestern University Feinberg School of Medicine, Chicago, USA; ^4^John A. Moran Eye Center, Department of Ophthalmology and Visual Sciences, Salt Lake City, UT, USA; ^6^0000-0002-2785-1716; ^7^0000-0003-2611-0526; ^8^The authors with asterisks contributed equally to this manuscript.

**Keywords:** Intrastromal Corneal Ring Segment, Keratoconus, Femtosecond Laser

## Abstract

**Purpose:**

To evaluate and compare clinical outcomes after femtosecond laser-assisted implantation of 325-degree versus 340-degree arc length intracorneal ring segments (ICRS) in eyes with keratoconus (KCN).

**Methods:**

In this prospective non-randomized interventional case series, 23 eyes of 21 patients diagnosed with KCN, underwent femtosecond laser-assisted implantation of two types of ICRS, which included a 325-degree ICRS (Group 325) and a 340-degree ICRS (Group 340). The primary outcome measures were uncorrected distance visual acuity (UDVA), and the secondary outcome measures included corrected distance visual acuity (CDVA), sphere, cylinder, mean refractive spherical equivalent (MRSE), keratometry, vectorial change in corneal astigmatism, and the location of maximum keratometry relative to the corneal apex. The study groups were compared using the primary and secondary outcome measures obtained at postoperative months six and 12.

**Results:**

Groups 325 and 340 consisted of 10 and 13 eyes, respectively. The two groups were comparable in terms of parameters measured preoperatively. On comparison to the baseline values, both study groups exhibited a significant increase in UDVA and CDVA measured at postoperative month six (*P*s < 0.05) and a significant decrease in the sphere, cylinder, spherical equivalent refraction, and keratometry readings measured at postoperative months six and 12 (*P*s < 0.05). No significant differences were observed between the two groups in terms of visual, refractive, and keratometric outcomes at any time point. No intraoperative or postoperative complications were observed in any of the study groups.

**Conclusion:**

Both the 325-degree ICRS and the 340-degree ICRS effectively and equally improved visual, refractive, and keratometric outcomes in keratoconic eyes.

##  INTRODUCTION

Keratoconus (KCN), the most common corneal ectatic disease, is characterized by progressive corneal thinning and bulging. Consequent myopia and irregular astigmatism result in reduced visual acuity. Recently, considerable advances have been made in the management of KCN. In the early stage of the disease, visual acuity can be restored with the use of glasses and rigid gas permeable (RGP) contact lenses. In more advanced cases with contact lens intolerance, the intrastromal corneal ring segment (ICRS) which renders the cornea flatter and more regular, can be implanted.^[1]^

ICRSs, usually made of polymethylmethacrylate (PMMA), are inserted deeply into the corneal stroma at the mid periphery. These segments flatten the central cornea through the arc-shortening effect, resulting in corneal remodeling.^[2,3]^ This treatment modality aims to reduce both myopia and irregular astigmatism and improve visual acuity, thereby postponing the need for keratoplasty.^[4,5,6,7]^

Various models with multiple designs and thicknesses have been introduced, since the introduction of corneal ring implants for myopia correction.^[8]^ Two main categories include complete continuous ring implants such as Myoring (Dioptex GmbH, Austria) or segmented implants termed ICRS such as Keraring (Mediphacos, Belo Horizonte, Brazil).^[9,10,11,12]^

The continuous ring implant acts as a second limbus and supports the cornea biomechanically. After continuous ring implantation, alterations in corneal shape occur in the area inside the ring, resulting in a flatter and more regular cornea.^[13,14,10,15]^ Furthermore, continuous rings implantations require the application of a simple nomogram and can be used for a wide range of keratoconus severity. On the contrary, short-arc ICRSs are appropriate for the less severe stages of the disease.

Keraring, (Keraring; Mediphacos, Belo Horizonte, Brazil) characterized by a triangular cross-section, is a commonly used ICRS for KCN. Its unique triangular cross-section reflects the incoming light rays through a prismatic effect and subsequently minimizes glare and halos.^[11]^ Various types of Keraring, differing in terms of thickness, arc length, and diameters are available and allow for customized correction of refractive errors.^[16,17]^ Long-arc ring segments have been introduced to exploit the primary benefits of continuous ring implants. In 2013, the 355-degree arc length Keraring was specifically proposed for nipple KCN. This ring segment can be implanted using the femtosecond laser.^[14]^ Although good results were obtained after implantation of the 355-degree arc length Keraring, some complications were reported.^[14,18]^ One study reported the corneal melt at the site of the incision as the most frequently encountered complication.^[19]^ This complication, which is attributable to the proximity between the ring tip and the incision site, remains the leading risk factor for extrusion, infection, and corneal melting.^[14]^ To reduce the odds of this complication, a 340-degree arc length Keraring (Keraring; Mediphacos) was proposed, which provided promising initial results.^[16]^ Subsequently, 340-degree arc length (AJL Ophthalmics, Vitoria, Spain) and 325-degree arc length (Keraring; Mediphacos) corneal rings were introduced to increase safety and preserve good outcomes.^[20,21]^ The main advantage of 340 and 325-degree arc length corneal rings is to provide a 15 and a 20-degree gap from the corneal incision on each side, respectively, which increases the safety profile. Despite these advantages, a decrease in the flattening effects of the reduced cord length of these rings, compared to the previous ones, remains a concern.^[22]^ To the best of our knowledge, no previous study has compared the efficacy of the 325 and 340-arc length Keraring in KCN affected eyes. In the present study, we aimed to compare the clinical outcomes of femtosecond laser-assisted implantation of 325-ICRS and 340-ICRS in keratoconic eyes.

##  METHODS

This comparative nonrandomized interventional case series was conducted on patients with a definite diagnosis of KCN who underwent 325 or 340-arc length Keraring implantation from March 2017 to March 2019. The study protocol was approved by the local Ethics Committee, affiliated with Shahid Beheshti University of Medical Sciences, and it adhered to the tenets of the Declaration of Helsinki. The patients were asked to provide written informed consent after a complete explanation of the study protocol. All surgeries were performed by the same surgeon (A.F.) at Negah Eye Hospital.

### Inclusion and Exclusion Criteria

KCN was diagnosed by an experienced cornea specialist, based on slit-lamp examination, topography, and dual Scheimpflug system (Galilei G4, Ziemer Ophthalmic Systems AG, Port, Switzerland). Inclusion criteria included ages between 20 and 40 years, stable disease over the past 12 months, unacceptable vision with spectacle correction, RGP contact lens intolerance, and minimum corneal thickness at the site of the ring implantation > 400 microns. In addition, patients who attended all the follow-up examinations up to postoperative month 12, were enrolled.

Exclusion criteria were mean keratometric (K) values > 65.0 dipoters (D), a history of previous ocular disease or corneal surgery, presence of central corneal opacity, corneal dystrophies, cataract, pregnancy or nursing, use of systemic medications that could affect the cornea (Isotretinoin, Amiodarone, Sumatriptan), and systemic collagen-vascular or autoimmune diseases. The KCN severity was graded based on the Amsler-Krumeich KCN classification as follows:^[23]^

Stage I: eccentric steeping; myopia or induced astigmatism of < 5.00 D, or both; and mean central K readings of < 48.00 D.

Stage II: myopia or induced astigmatism from 5.00 to 8.00 D, or both; mean central keratometry readings of < 53.00 D; absence of scarring; and minimum corneal thickness of > 400 microns.

Stage III: myopia or induced astigmatism from 8.00 to 10.00 D, or both; mean central keratometry readings of > 53.00 D; absence of scarring; and minimum corneal thickness between 300 and 400 microns.

Stage IV: non-measurable refraction, mean central keratometry readings of > 55.00 D, central corneal scarring, and minimum corneal thickness of < 200 microns.

**Figure 1:**
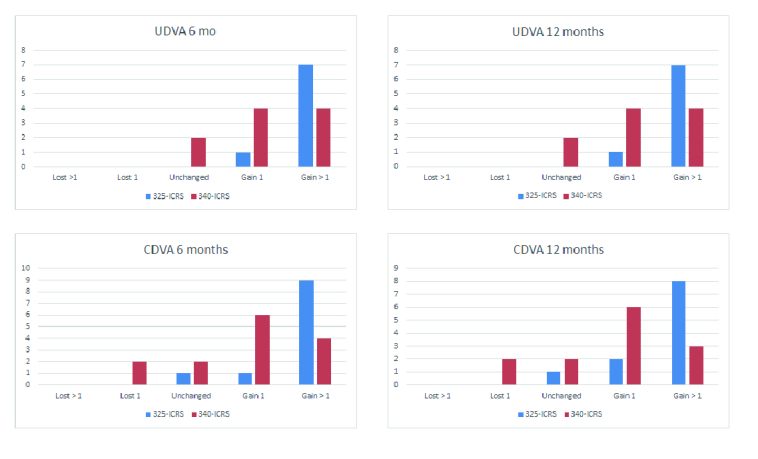
Corrected distance visual acuity (CDVA) changes 12 months postoperatively.

**Figure 2:**
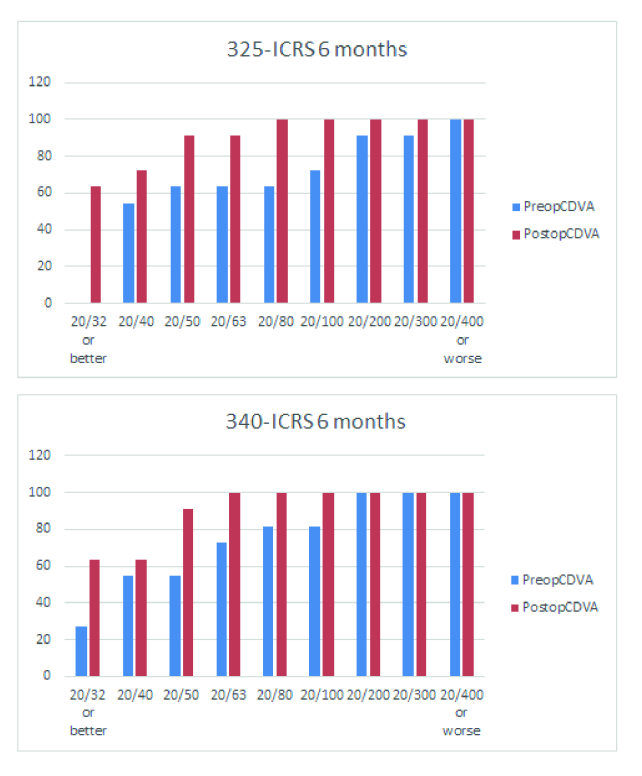
Safety charts. Cumulative measures of postoperative corrected distance visual acuity were plotted against preoperative corrected distance visual acuity.

**Figure 3:**
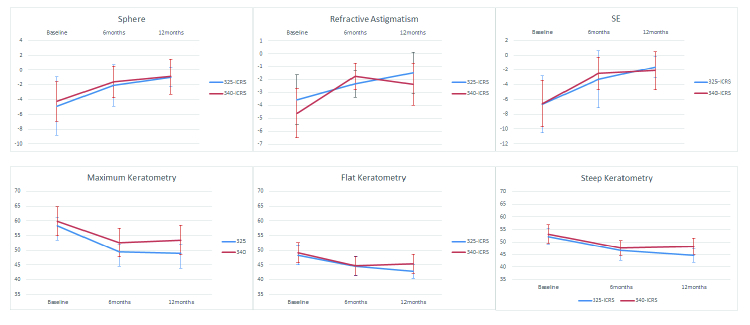
Line charts plotting refractive and keratometric outcomes of study groups. Blueline represents 325-ICRS while Redline depicts 340-ICRS.

**Figure 4:**
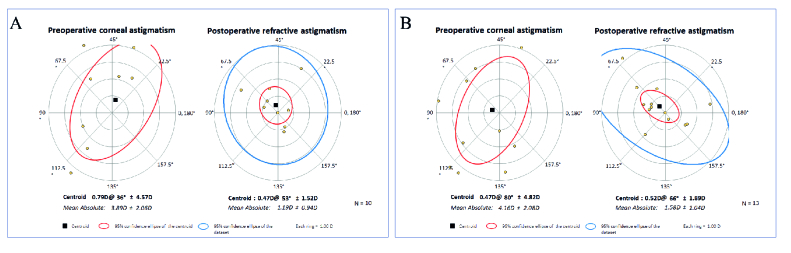
Double angle plots in A) 325 ICRS group, and B) 340 ICRS group.

**Table 1:** Comparison of baseline characteristics between the study groups.


orangeParameter	orangeGroup 1 (10 Eyes)	orangeGroup 2 (13 Eyes)	orangeP-Value
Mean age (y) ± SD	31 ± 4.94	29 ± 4.26	0.277 ‡
Sex, n (%)		
Male	7 (70.0 %)	5 (38.5 %)	
Female	3 (30.0 %)	8 (61.5 %)	0.374*
Eye, n (%)		
Right	3 (30.0%)	7 (53.8%)	
Left	7 (70.0%)	6 (46.2%)	0.688*
Keratoconus Grading		
Stage I, n (%)		
Stage II, n (%)	1 (10%)	1 (7.69%)	
Stage III, n (%)	6 (60%)	8 (61.53%)	0.456*
Stage IV, n (%)	3 (30%)	1 (7.69%)	
	0 (0.0%)	3 (23.07%)	
	
	
white<bcol>4</ecol> ‡ Based on T-test; * Based on Chi-squared test. SD, standard deviation

**Table 2:** Refractive outcomes of two study groups over the study period.


	orange**Group 1 (ring 325)**	orange**Group 2 (ring 340)**	orangeP-Value*
Mean sphere (D)		
Preoperative	- 4.9 ± 3.94	- 4.26 ± 2.67	0.69
Month 6	- 2.1 ± 2.82	- 1.59 ± 2.17	0.358
P-value ** ${}^{\gamma }$ **	0.044	0.002	
Month 12	- 0.97 ± 1.3	- 0.90 ± 2.39	0.216
P-value ** ${}^{\gamma }$ **	0.037	0.013	
Mean corneal astigmatism (D)		
Preoperative	- 3.57 ± 2.12	- 4.61 ± 1.92	0.12
Month 6	- 2.35 ± 2.74	- 1.78 ± 1.05	0.68
P-value ** ${}^{\gamma }$ **	0.028	0.003	
Month 12	- 1.5 ± 1.2	- 2.38 ± 1.63	0.42
P-value ${}^{\gamma }$	0.048	0.041	
Mean refractive SE (D)		
Preoperative	- 6.68 ± 3.82	- 6.57 ± 3.15	0.94
Month 6	- 3.27 ± 3.89	- 2.49 ± 2.21	0.192
P-value ** ${}^{\gamma }$ **	0.041	0.005	
Month 12	- 1.63 ± 1.41	- 2.10 ± 2.59	0.151
P-value ** ${}^{\gamma }$ **	0.017	0.014	
	
	
white<bcol>4</ecol>* ** ${}^{\gamma }$ ** Based on linear mixed model; Multiple comparisons are corrected using the Bon-Ferroni method. SE, Spherical Equivalent

### Preoperative Assessment

An ophthalmic examination including slit-lamp biomicroscopy, Goldmann applanation tonometry, and dilated fundus examination was performed in all patients. Uncorrected distance visual acuity (UDVA), corrected distance visual acuity (CDVA), sphere, cylinder, and mean refractive spherical equivalent (MRSE) were measured. Computerized corneal topography (TMS, Tomey GmbH, Nagoya, Japan) was performed to determine the steep keratometry, flat keratometry, mean keratometry (Kmean), and maximum keratometry (Kmax). The root of the sum of squares of horizontal and vertical distances of the point with maximum keratometry was calculated from the corneal apex in each study group. In addition, corneal tomography was performed using a dual Scheimpflug system to determine the cone location, corneal thickness at the implantation zone, and the incision site.

### Surgical Technique

All procedures were conducted under topical anesthesia. In all the cases, corneal tunnels were created using a femtosecond laser (FemtoLDV Z6, Ziemer Ophthalmic System AG, Port, Switzerland). Centration was obtained by asking the patient to look at the central red light of the femtosecond laser machine, and after docking, the location of the tunnel was adjusted according to the pupillary margin. The femtosecond laser was used to create a tunnel with the following parameters; inner diameter: 5.4 mm, outer diameter: 6.6 mm; and depth: 75% of the thickness of the thinnest point along with the proposed tunnel site. The incision leading to the tunnel was made on the steep meridian. The ring thickness was chosen based on the MRSE and corneal thickness. Both 325-ICRS and 340-ICRS were available in 5.0 mm internal diameter, 6.4 mm outer diameter, and 700 microns at the base. The 340-degree ICRS was available in 2 thicknesses including 200 microns recommended for eyes with an MRSE of < – 6.00 diopters, and 300 microns for an MRSE of ≥ - 6.00D. The thickness of the 325-ICRS varied between 150 and 350 microns with a 50-micron increment.^[21]^ The same recommendation was followed to choose the thickness of the 325-ICRS (200 microns for an MRSE of < – 6.00 diopters and 300 microns for an MRSE of ≥ - 6.00D). The device was implanted through the incision and rotated in the tunnel until its tips were at an equal distance from the incision site. At the end of the procedure, a soft bandage contact lens was placed, and chloramphenicol eye drop was instilled.

### Postoperative Assessment

Patients were examined on postoperative days 1 and 7 and at postoperative months 1, 3, 6, and 12. Complete eye evaluation, including UDVA, CDVA, slit-lamp biomicroscopy, Goldmann applanation tonometry, and corneal imaging by Placido disc topography and dual Schiempflug tomography, was performed at postoperative months 6 and 12.

### Statistical Analysis

Data were presented as means, standard deviations, medians, ranges, and frequencies. Demographic data were compared between the study groups using Mann-Whitney, Chi-Square, and Fisher's exact tests. A linear mixed model with an interaction term of the follow-up time and groups were performed to compare the outcome between and within the groups.

All statistical analyses were performed using the SPSS statistical software (IBM Corp. Released 2017. IBM postoperatively, SPSS Statistics for Windows, Version 25.0. Armonk, NY: IBM Corp.). All *P*-values were two-sided, and a *P*-value of < 0.05 was considered statistically significant. Vector analysis was performed with Alpin's method using astigmatic software and data sets (can be downloaded from http://www.lasikmd.com/media/astigmatic)

##  RESULTS 

This study enrolled 23 eyes of 21 patients; 52.2% of the patients were men. The mean patient age was 29.86 ± 4.57 years (median = 31, range 20 to 36 years). The 325-ICRS and 340-ICRS were implanted in 10 and 13 eyes, respectively. Table 1 compares demographics and preoperative parameters between the study groups. Two study groups were comparable regarding preoperative visual acuity, refractive error, keratometry readings, and KCN severity.

### Visual Outcome

Compared to the baseline values, the postoperative UDVA was significantly improved in both groups. In the 325-ICRS group, the mean UDVA increased from 1.18 ± 0.17 logMAR preoperatively to 0.56 ± 0.27 logMAR (*P* = 0.011) and 0.53 ± 0.23 logMAR (*P* = 0.012) measured at postoperative months six and 12, respectively. In the 340-ICRS group, the mean UDVA was 1.01 ± 0.13 logMAR at baseline, which increased to 0.57 ± 0.29 logMAR (*P* = 0.018) and 0.55 ± 0.28 logMAR (*P* = 0.019) measured at postoperative months six and 12, respectively. Compared to the baseline values, the mean CDVA was significantly improved in both study groups from postoperative month six onward in each study group; both groups were comparable in terms of the postoperative CDVA measured at each time point [Supplementary Table 1].

Figure 1 shows gains in CDVA in the study groups. No eyes lost any Snellen line, whereas 87.1% and 57.1% of the eyes gained 1 or more Snellen lines in 325-ICRS and 340-ICRS groups, respectively. Figure 2 presents the safety of 325 -ICRS and 340-ICRS.

### Refractive Outcome

The baseline mean sphere was - 4.9 ± 3.94 D and - 4.26 ± 2.67 D in 325-ICRS and 340-ICRS groups, respectively (*P* = 0.69). This value was significantly decreased to - 1.59 ± 2.17 D at postoperative month six (*P* = 0.002) and - 0.9 ± 2.39 D at postoperative month 12 (*P* = 0.01) in the 340-ICRS group. Similarly, in the 325-ICRS group, the mean sphere measured at two-time points ( - 2.1 ± 2.82 D, *P* = 0.04 and - 0.97 ± 1.31 D, *P* = 0.04, respectively) was significantly reduced, as compared to the baseline value. The two groups were comparable in terms of the mean sphere measured at postoperative months six and 12 (*P* = 0.35 and 0.21, respectively).

At baseline, the mean refractive astigmatism was - 3.57 ± 2.12D and - 4.61 ± 1.92D in 325-ICRS and 340-ICRS groups, respectively (*P* = 0.12). In the 340-ICRS group, the mean MRSE was significantly decreased to - 2.35 ± 2.74D (*P* = 0.028) and - 1.5 ± 1.2D (*P* = 0.048) measured at postoperative months six and 12, respectively. The corresponding values were -1.78 ± 1.05D (*P* = 0.003) and - 2.38 ± 1.63D (*P* = 0.041) in the 325-ICRS group, respectively. The two groups were comparable in terms of MRSE measured at all time points (*P* = 0.12 at baseline, 0.68 at postoperative month six, and 0.42 at postoperative month 12) [Table 2].

### Keratometric Outcome

Supplementary table 2 presents the keratometric results. The mean preoperative Kmax was 57.6 ± 2.1 D in the 325-ICRS group and 58.9 ± 5.1 D in the 340-ICRS group (*P* = 0.37). Six months after the operation, this measurement decreased to 49.9 ± 2.8 D (*P* = 0.005) and 52.1 ± 5.1 D (*P* = 0.006) in groups 1 and 2, respectively. The corresponding values measured at postoperative month 12 were 48.97 ± 2.94 D (*P* = 0.012) and 53.47 ± 5.1 D (*P* = 0.017), respectively. No statistically significant differences between the study groups in the postoperative Kmax at any time point (*P* = 0.14 and 0.07 at postoperative months six and 12, respectively).

In the 325-ICRS group, the average of Kmean was 50.2 ± 3.3D before the operation that was significantly decreased to 45.1 ± 3.6 D (*P* = 0.004) and 44.60 ± 2.72 D (*P* = 0.001) after six and 12 months, respectively. The corresponding values in the 340-ICRS group were 49.9 ± 3.4D at baseline, 45.5 ± 2.9D at month six (*P* = 0.001), and 45.6 ± 1.2D at month 12 (*P* = 0.003). The two groups were similar in terms of Kmean at all time points (*P* = 0.45, 0.87, and 0.24 at baseline, and postoperative months six and 12, respectively).

Intragroup and intergroup analyses failed to demonstrate significant differences at any time point in the root of the sum of squares of horizontal and vertical distances of the maximum keratometry from the corneal apex. Figure 3 presents a summary of the refractive and keratometric outcomes of the study groups.

### Vector Analysis 

The mean target-induced astigmatism was 2.7 ± 1.8 and 4.42 ± 1.5 D in 325-ICRS and 340-ICRS groups, respectively (*P *= 0.08). The mean surgically induced astigmatism (SIA) was 3.87 ± 1.19 and 3.71 ± 1.5 in 325-ICRS and 340-ICRS groups, respectively (*P* = 0.26). The two groups were comparable in terms of target-induced astigmatism, surgically induced astigmatism, correction index, the magnitude of error, and angle of error [Figure 4].

### Complications

No intraoperative or postoperative complications were observed during the follow-up period in the study groups. Two representative figures and presentation of patients are depicted in supplementary figures 1 and 2.

##  DISCUSSION 

Results of the present study demonstrated the midterm clinical outcomes of the femtosecond laser-assisted implantation of 325-degree and 340-degree arc length ICRSs in keratoconic eyes. To reduce the postoperative glare and halo, the tunnel was adjusted to the pupillary margin rather than to the cone. We found a significant reduction in the postoperative sphere, cylinder, and MRSE in both study groups. In addition, keratometric parameters were significantly reduced, indicating central corneal flattening after ring implantation in both groups. The improvement of visual and keratometric values remained stable for at least one year.

Although the primary objective of the long-arc ICRS was to increase the flattening effect of the rings, particularly for the nipple type of keratoconus, we utilized them for both central and paracentral types of keratoconus. Our results in the 340-ICRS group were in line with the previous studies reporting the results of near-complete ICRS implantation.^[24,25,14]^Sadoughi et al^[25]^ implanted a 340-ICRS in 18 eyes of patients with KCN and reported improvement in visual, refractive, and keratometric measurements. In their study, the mean sphere, cylinder, and MRSE were decreased by 3.4 D, 3.1 D, and 5.0 D, respectively. Corresponding values were 3.4 D, 2.2 D, and 4.5 D in the 340-ICRS group of the present study.

Our results in the 325-ICRS group were comparable with the results of previous studies implanting the same type of ICRS. Yousif et al^[26]^ investigated the efficacy and safety of three different rings implanted in 73 keratoconic eyes. One of the study groups consisted of 320-degree ICRS implantation. They reported that the mean MRSE decreased significantly by 4.34 D after six months, which was comparable to the value achieved in the present study (4.5 D). Similarly, Rocha et al^[27]^ analyzed the results in 34 eyes that underwent 320-degree arc length Ferrara ring segment implantation (AJL Ophthalmics, Vitoria, Spain) and reported that UDVA, CDVA, MRSE, and all corneal tomographic parameters significantly improved after the operation. Torquetti et al conducted a multicenter nonrandomized study in which a 320-degree arc length Ferrara (AJL, Vitoria, Spain) ICRS was implanted in 138 eyes of 130 patients with KCN. The average of Kmean and MRSE were reduced postoperative, by approximately 5.5 D and 3.75 D, respectively.^[19]^ The authors concluded that the flattening effect of this implant is greater in the steep corneas as compared to the flat corneas. However, we did not observe this effect in the present study. The preoperative mean keratometry in the present study was at least 3.0 D lower than that reported in their study; however, the Kmean and MRSE in our study decreased by 6.0 and 5.0 D, respectively, which were slightly better than those reported by Torquetti et al. Another reason explaining the difference could be attributed to the difference in the base diameter of the ICRS (700 microns in the present study vs 600 microns in Toerquetti et al).

One can hypothesize that the larger the ICR arc length is, the greater is the flattening effect. However, our results demonstrate comparable efficacy with both 325- and 340-ICRS implantation. This finding can be attributed to the small sample size in the present study.

Abdallah et al^[24]^ investigated the results of the femtosecond laser-assisted implantation of 355-ICRS and reported that postoperative sphere, cylinder, and spherical equivalent (SE) were reduced by 3.36 D, 2.00 D, and 4.3 D, respectively; these results were in agreement with those we achieved in both the 340-ICRS and 325-ICRS groups. However, Jadidi et al^[14]^ reported slightly lower values of improvement after implantation of a 355-degree ICRS using a pocket maker; the mean reduction in the sphere, cylinder, and MRSE was 2.13 D, 2.4 D, and 3.45 D, respectively, in their study. This lower effect might be due to implanting of the ring in a pocket rather than a tunnel, which probably decreased the flattening effect of the ICRS. The flattening effect of the 355-degree arc length Keraring ICRS in one study conducted by Abd Elaziz et al was better than the present study.^[18]^ They analyzed the results of 355-degree arc length ICR implantation within tunnels created by femtosecond laser in 30 eyes with advanced central KCN and reported a reduction of 6.2, 4.1, and 7.6 D in the postoperative sphere, cylinder, and MRSE, respectively; these results were better than those we achieved in this study.

Sadoughi et al^[25]^ found that the preoperative K value had a negative correlation with postoperative CDVA, and reported that the postoperative CDVA was lower in patients with Amsler-Krumeich grade IV disease. Similarly, Alio et al^[28]^ reported that eyes with the mean K values of ≤ 53.0 D had better visual outcomes than those with the mean K values of > 55.0 D after Intacs ICRS implantation. Nonetheless, several reports have confirmed the effectiveness of the Keraring and Ferrara ring implantation in the severe form of KCN.^[18,26]^ In our study, no statistically significant differences were observed in postoperative visual outcomes, MRSE, and keratometry measurements in different stages of KCN. However, the generalizability of this finding is limited by the small sample size.

To the best of our knowledge, the current study was the first that compared visual and keratometric outcomes after implantation of 325- and 340-degree arc length Keraring ICRs using a femtosecond laser. One limitation of the current study was the small sample size. This could affect the distribution of patients with different KCN severity between the two groups. Randomized controlled studies with larger sample size are required to verify our results. The second limitation lies in the fact that we did not evaluate and compare the effect of the implantation of these two rings on higher-order aberrations. Another drawback of our study was the inclusion of both eyes of two patients. Due to the small sample size, it was estimated that generalized estimating equation would not work as the best analysis method to correlate for inter-eye correlation. Therefore, we used the linear mixed model that could outperform generalized estimating equation in this situation.^[29]^

In summary, the results of the present study demonstrated that implantation of both 325- and 340-degree arc length Keraring ICRs comparably reduced keratometric indices. We did not find any significant association between the preoperative mean K values and the postoperative CDVA in any group.

##  Financial Support and Sponsorship

None.

##  Conflicts of Interest

None.
